# Supplementary material for: A neuronal correlate for time interval estimation in the crow’s telencephalon
Source: Nat Commun. 2025 Sep 10;16:8256. doi: 10.1038/s41467-025-63820-5 (PMC12423286; doi:10.1038/s41467-025-63820-5)
Supplement: Supplementary file 1 — Supplementary Information [file 41467_2025_63820_MOESM1_ESM.pdf]

A neuronal correlate for time interval estimation in the crow's telencephalon

Melissa Johnston[#], Maximilian E. Kirschhock[#], & Andreas Nieder*

Animal Physiology Unit, Institute of Neurobiology, University of Tübingen, 72076 Tübingen, Germany

# These authors contributed equally

* Correspondence: andreas.nieder@uni-tuebingen.de

ORCID iD: Andreas Nieder (0000-0001-6381-0375)

Supplementary Information

Supplementary Figures 1–4

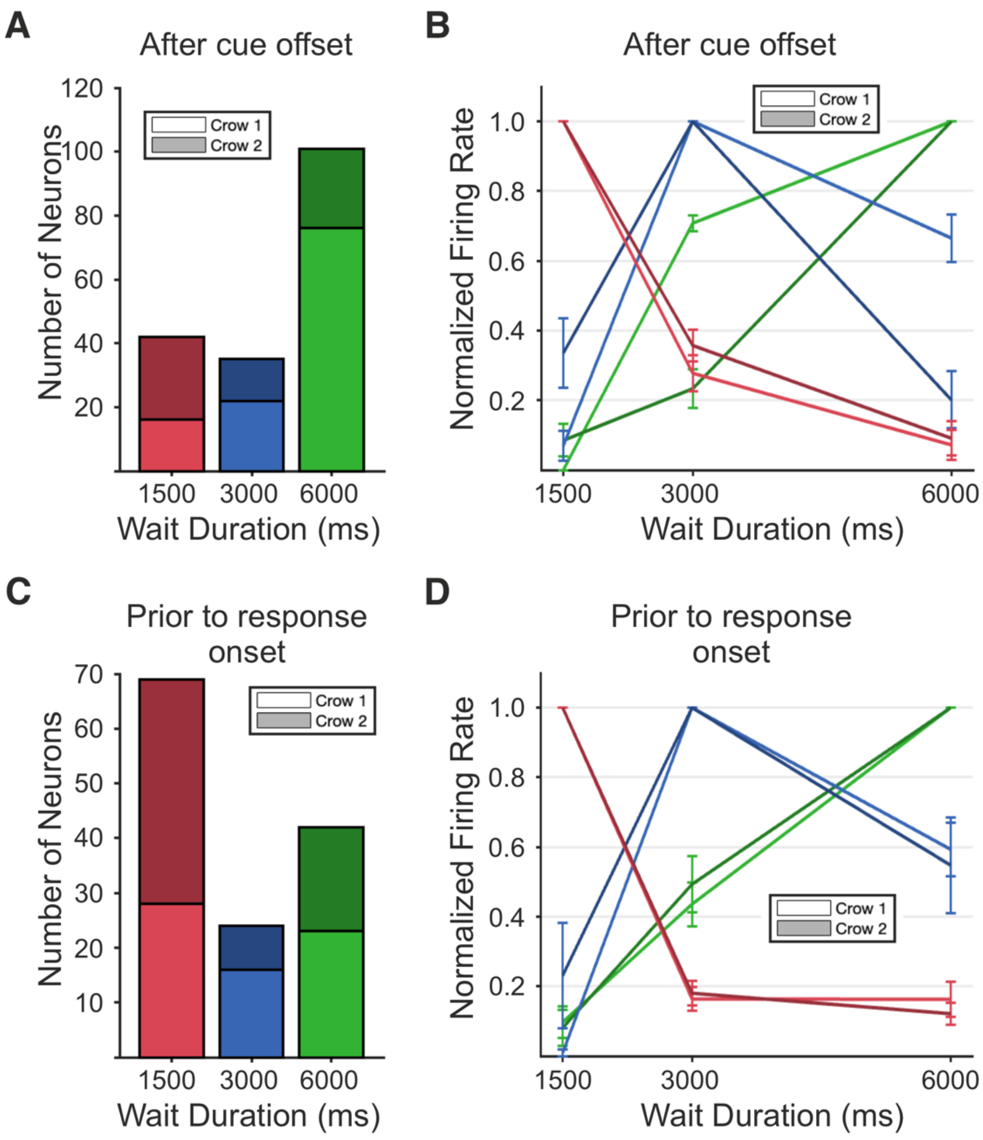

Supplementary Figure 1. Target wait preference and population tuning curves separated by crow. (A,C) Incidence of neurons preferring either the 1500, 3000, or 6000 ms target duration a 1200 ms period aligned to either after the cue offset **(A)** or prior to response onset **(C). (B,D)** Population tuning curves for neuronal activity aligned to after the cue offset (n = 178) **(B)** and prior to response onset (n = 135) **(D)**. Curves were obtained by averaging the normalized tuning curves of all single units selectively preferring either the 1500, 3000, or 6000 ms wait duration from **(A)** and **(C)**. Error bars show the standard error of the mean.

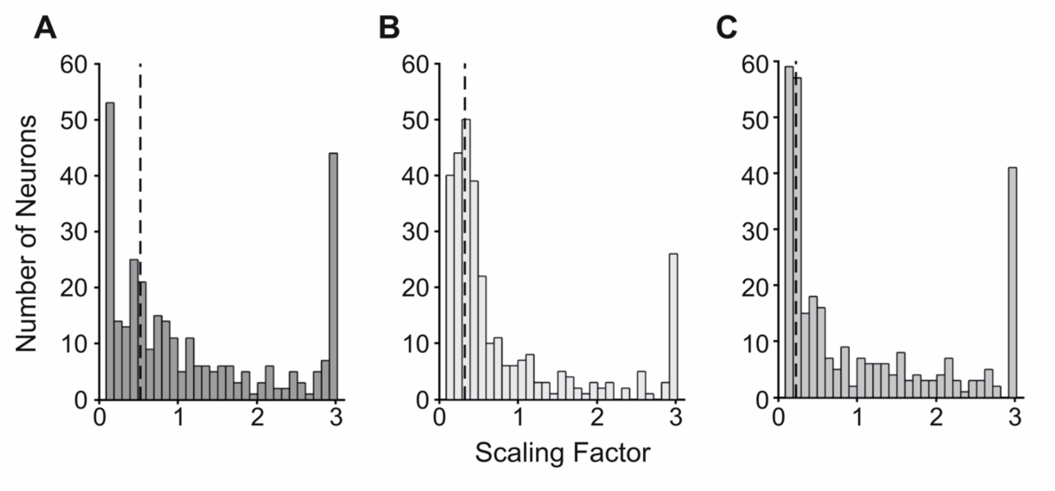

Supplementary Figure 2. **Scaling of individual neurons from longer to shorter wait durations (n = 409).** Neuronal activity was arranged into 10 ms bins and scaling was done using various scaling factors (0.3–3 in steps of 0.25) and calculated the associated mean squared error. The optimal scaling factor was that with the smallest difference (mean squared error) between the scaled and scaled-to activity, with larger scaling factors indicating less scaling. We compressed the first 2400 ms of data from 3000 ms trials to 1200 ms **(A)**, as well as the first 4800 ms of activity from 6000 ms trials to either 2400 **(B)** and 1200 ms **(C)**. Dashed line represents mean of minimal (or optimal) scaling factor.

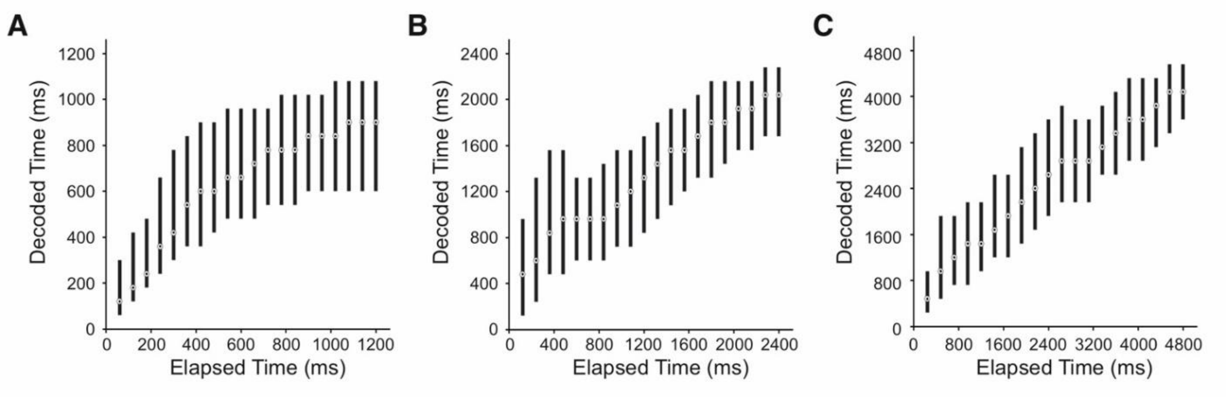

Supplementary Figure 3. Decoding of elapsed time from all neurons. (A–C) Decoding of elapsed time from the binned activity of all neurons (n = 409) aligned to cue offset. Neural activity was divided into 20 equal-sized bins for the first 1200 ms of the 1500 ms trials (60 ms; **A**), 2400 ms of the 3000 ms trials (120 ms; **B**), and 4800 ms of the 6000 ms trials (240 ms; **C**). This period represents the earliest response that was considered correct. Dot represents the median and the bar represents the interquartile values.

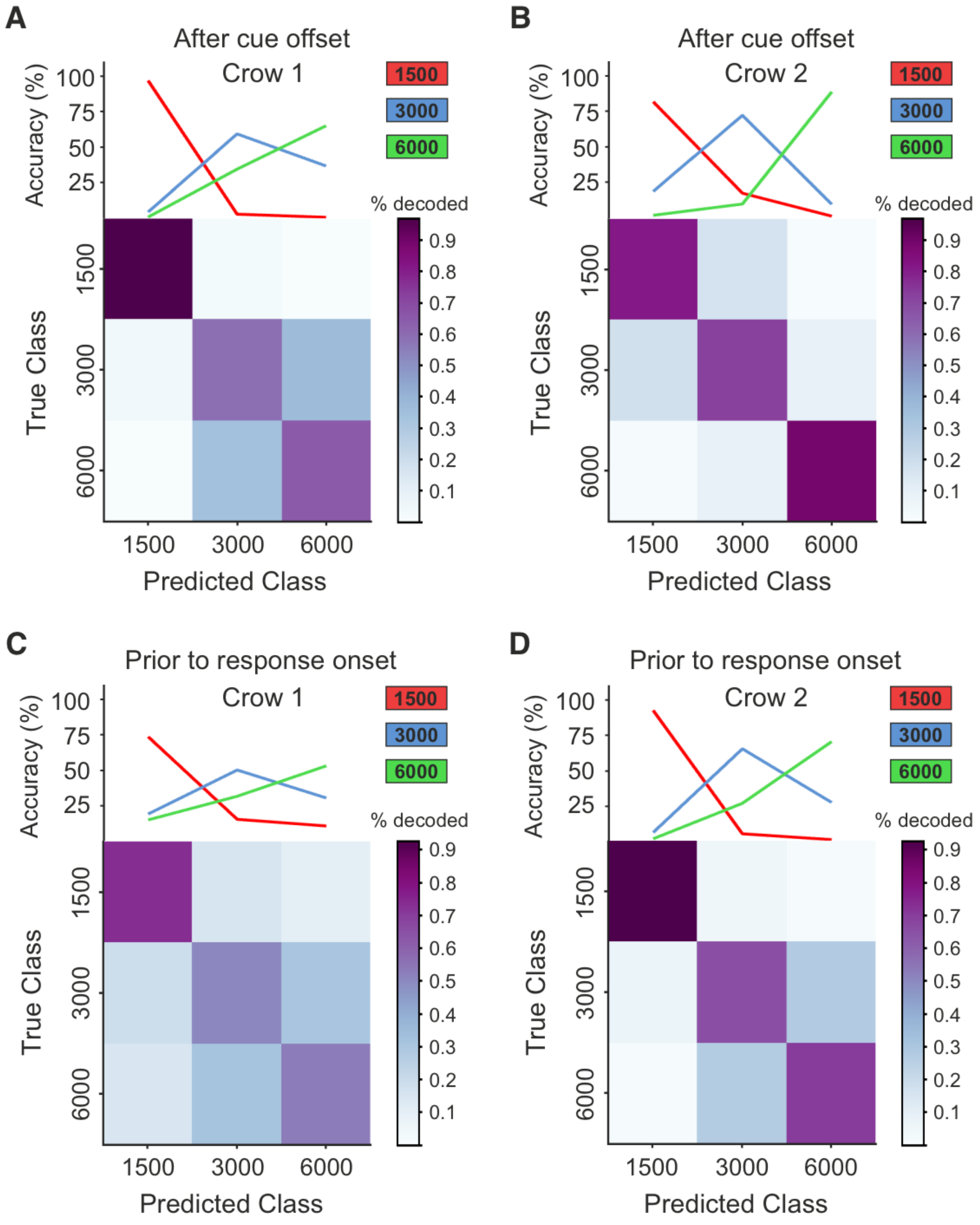

Supplementary Figure 4. Population decoding of target duration separated by crow. Linear multiclass support vector machine (SVM) classifiers using 1200 ms of neuronal activity aligned to either the cue offset **(A,B)** or response onset **(C,D)**. Accuracy of within- (trained on trials from one stimulus protocol and tested on new trials from the same protocol) and across-stimulus protocol performances (trained on trials from one stimulus protocol and tested on new trials from the respective other protocol) classifier prediction performance **(A,C)**. Performance of SVM classifiers target duration **(B,D)**. Top panels show classification performance for each target duration. Bottom panels depict the confusion matrices (averaged over 10-fold cross-validation and 1000 resamples). The scaling of colormaps is the same across both task periods. Crow 1: n = 265; Crow 2: n = 144.
